# The m6A methyltransferase METTL3 affects autophagy and progression of nasopharyngeal carcinoma by regulating the stability of lncRNA ZFAS1

**DOI:** 10.1186/s13027-021-00411-1

**Published:** 2022-01-03

**Authors:** Jiaojiao Peng, Hong Zheng, Feng Liu, Qi Wu, Shixi Liu

**Affiliations:** Department of Otolaryngology, Head and Neck Surgery, West China Hospital, Sichuan University, No. 37, Guoxue Lane, Chengdu, 610041 Sichuan China

**Keywords:** Nasopharyngeal carcinoma, lncRNA ZFAS1, N6-methyladenosine, Autophagy

## Abstract

**Background:**

Nasopharyngeal carcinoma (NPC) is a malignant tumor originating from the epithelial cells of the nasopharyngeal mucosa of the head and neck. The role of long non-coding RNA and RNA methylation in NPC has received increasing attention. Therefore, this study aims to investigate the mechanism of lncRNA ZFAS1 in NPC and its relationship with RNA methylation, providing evidence for targeted therapy of NPC.

**Methods:**

Microarray arrays were used to screen the differentially expressed miRNAs in normal tissues and tumor tissues. QRT-PCR was used to quantify ZFAS1, miR-100-3p, ATG10, autophagy and epithelial-mesenchymal transition related genes. The interactive relationship between ZFAS1 and miR-100-3p was verified using dual-luciferase reporter gene assay and RIP assay. CCK-8, transwell and apoptosis were used to detect the occurrence of tumor cells after different treatments. The m6A modification test is used to verify the effect of METTL3 on ZFAS1. BALB/c mice and BALB/c nude mice are used to detect the effects of different treatments on tumor growth and immune escape in vivo.

**Results:**

ZFAS1 is upregulated in tumor tissues and NPC cells. N (6)-methyladenosine (m6A) is highly enriched in ZFAS1 and enhances its RNA stability. ZFAS1 is used as an oncogenic lncRNA, which can promote NPC cell proliferation, migration and tumor growth. In terms of mechanism, ZFAS1 up-regulates the expression of ATG10 by competitively adsorbing miR-100-3p and regulates the level of autophagy by inhibiting the PI3K/Akt signaling pathway to promote the proliferation and migration of NPC cells.

**Conclusion:**

In short, our study verified the cancer-promoting effect of ZFAS1 in NPC and explained part of the reason for its upregulation. In addition, we confirmed that ZFAS1 can regulate the autophagy level of NPC cells through the PI3K/AKT pathway through miR-100-3p/ATG10 to affect tumor progression.

**Supplementary Information:**

The online version contains supplementary material available at 10.1186/s13027-021-00411-1.

## Background

Nasopharyngeal carcinoma (NPC) is a malignant tumor derived from the epithelial cells of the nasopharyngeal mucosa of the head and neck. In the nasopharynx, tumors are usually observed in the pharyngeal crypts [1]. In 2018, there were 129,000 new cases of cancer in the world, accounting for 0.7% of the global incidence of cancer. Its prevalence is unevenly distributed globally, with most NPC patients mainly located in Southeast Asia, while China has one of the highest morbidity and mortality rates in the world [2]. Although the current radiotherapy and chemotherapy methods are widely used in the treatment of NPC, like most tumors, the stage of the disease is a key factor affecting prognosis and survival [3]. The latest data shows that only early radiotherapy for NPC has the best effect, but as the lesion progresses, local and distant recurrence will affect the 5-year overall survival rate. The overall survival rate for stage I and stage II diseases has dropped from about 90% to 75% [3]. Although we have realized the importance of early detection, like most tumors, NPC has no obvious symptoms in the early stage, which often leads to misdiagnosis, treatment delays and adverse outcomes [4]. Therefore, finding efficient biomarkers for early diagnosis and in-depth exploration of pathogenic mechanisms, effective development of disease prevention and targeted therapy is the top priority of current research.

Non-coding RNAs refer to RNAs that do not code for proteins. Common ones include microRNAs (miRNAs), long non-coding RNAs (lncRNAs) and circRNAs, etc. [5]. A large number of studies have reported that lncRNA and miRNA are highly correlated with various tumors. Current researches often focus on the mechanisms of lncRNA and miRNA in the development of NPC, radiosensitivity, chemotherapy resistance and angiogenesis [6–8]. For example, lncRNA CASC2 can inhibit the proliferation of NPC and induce apoptosis by inhibiting the activation of miR-18a-5p/RBBP8 axis [9]. The high expression of miR-7 inhibits the proliferation and invasion of NPC cells by down-regulating the expression of MPC-7 [9]. This research group proved that lncRNA ZFAS1 can promote the proliferation and metastasis of NPC through miR-892b/LPAR1. Furthermore, the effect of ZFAS1 was also observed in NPC cells resistant to radiotherapy. However, the reason for its differential expression and the targeting mechanism are not clear.

N6-methyladenosine (N6-methyladenosine, m6A), the methylation of adenylate (A) at the sixth position in RNA, is the most common way of RNA modification [10]. This process is dynamically and reversibly regulated by methyltransferases (Writers) and demethylases (Erasers), and by binding to m6A readers (Readers), it affects the stability of mRNA, regulates gene expression and determines cell growth, differentiation [11]. Methyltransferase is a multi-component complex composed of METTL3, METTL14, RBM15B and ZC3H13, among which the main catalytic function is performed by METTL3 [12]. Studies have shown that the m6A modification reported in the mammalian genome can directly recognize the m6A site to regulate RNA expression, splicing, translocation, lncRNA stability, miRNA processing, circRNA loop formation and RNA–protein interaction and other ways to affect RNA function [12, 13]. Recent studies have shown that the abnormal expression of m6A modified RNA could promote the growth and survival of cancer cells, as well as the occurrence and development of tumors. A study on m6A methyltransferase showed that knocking down METLL3 or METLL14 can promote the stemness and tumorigenesis of glioblastoma stem cells [14]. In the study of NPC, it was found that METTL3 can promote the occurrence and development of tumors by stabilizing the expression of lncRNA FAM225A [13].

In this study, we found that the high expression of ZFAS1 is closely related to the poor prognosis of patients with NPC. The m6A marker improve the stability of methylated ZFAS1 transcripts by reducing the rate of RNA degradation, which may be part of the reason for the up-regulation of ZFAS1 in NPC. In vitro and in vivo functional experiments show that ZFAS1 promotes the proliferation and metastasis of NPC cells by regulating the level of autophagy. ZFAS1 is a competitive endogenous RNA (ceRNA), sponge miR-100-3p increases the expression of ATG10, and regulates the PI3K/Akt/mTOR pathway to affect the level of autophagy. Our study elucidates the clinical significance and regulatory mechanism of ZFAS1 in NPC and provides a prognostic indicator as well as a promising therapeutic target for NPC patients.

## Materials and methods

### Clinical sample

Collected tumor tissues and matched adjacent tissues from 53 NPC patients admitted to West China Hospital of Sichuan University from January 2018 to December 2019. All cases were diagnosed by histopathological examination. None of them were treated with chemotherapy or radiotherapy. This study was approved by the Institutional Ethical Review Board of the Sichuan University West China Hospital, and written informed consent was obtained from all patients. The clinicopathological features of participants were listed in Additional file 5: Table S1. The 53 cases of nasopharyngeal carcinoma tissue and para-cancerous tissue specimens were collected strictly in accordance with the specimen collection specifications during the operation, some were frozen and stored at − 80 °C, some were fixed with 10% formalin, and dehydrated by an automatic dehydrator. Embed in paraffin for preservation.

### Cell culture

We used ten NPC cell lines including HONE-1, CNE-1, CNE-2, HNE-1, C666-1, HK-1, S26, S18, SUNE-1 and 6-10B. The human immortalized nasopharyngeal epithelial cell lines (NP69 and N2Tert) served as a control. They were collected from the Cellbank of the Chinese Academy of science (Shanghai, China). Cells were grown routinely in RPMI-1640 medium (Invitrogen, Carlsbad, CA, USA) with 10% fetal bovine serum (Gibco, Carlsbad, CA, USA) and cultured in a 37 °C humidified atmosphere with 5% CO_2_.

### Microarray analysis

MiRNA expression was analyzed using the Arraystar Human miRNA Microarray v3.0 by KangChen Bio-tech as previously described. Strict screening criteria were used to identify differentially expressed miRNAs(log2FC > 2, FDR < 0.05). Heat map and volcano plots were generated with the "pheatmap" and "ggplot2" packages in R (version 3.4.4).

### GEO database analysis

The data set used is from GEO database (https://www.ncbi.nlm.nih.gov/geo/), and the download data format is MINIML (NO: GSE12452, GSE64634). The m6A-related genes are derived from Juan Xu's research on the molecular characterization and clinical significance of m6A modulators across 33 cancer types. PCA graphs are drawn by R software package ggord; The box plot is implemented by the R software package ggplot2; the heat map is displayed by the R software package pheatmap. All the above analysis methods and R package were implemented by R foundation for statistical computing (2020) version 4.0.3.

The extracted data were normalized and processed by log2 transformation. the microarray data were normalized using the preprocessCore package in R software (version 3.4.1). Probes were converted to gene symbols according to the platform annotation information of the normalized data. Probes with more than one gene were eliminated and the average value was calculated for genes corresponding to more than one probe. As an initial quality control step using variance stabilized counts with individual horse effect removed using the removeBatchEffect function of limma R package.

### The Cancer Genome Atlas (TCGA) database analysis

Raw counts of RNA-sequencing data (level 3) and corresponding clinical information from 62 NPC samples were obtained from The Cancer Genome Atlas (TCGA) dataset (https://portal.gdc.cancer.gov/) in January 2020, in which the method of acquisition and application complied with the guidelines and policies. The m6A-related genes are derived from Juan Xu's research on the molecular characterization and clinical significance of m6A modulators across 33 cancer types.

All the above analysis methods and R package were implemented by R foundation for statistical computing (2020) version 4.0.3 and software packages ggplot2 and pheatmap.

### Plasmid construction and cell transfection

On the GenePharma (Shanghai, China), the transfected materials miR-100-3p mimics and inhibitor, si-METTL3-1,2, oe-ZFAS1 and sh-ZFAS1 were purchased. Twenty-four hours before transfection, HK-1 and HONE-1 cells in the exponential phase were digested by pancreatin and made into cell suspension. miR-100-3p mimics and inhibitor, si-METTL3-1,2, oe-ZFAS1 were used for cell experiment and the in vitro experiment. After trypsinization from flasks, cells were cultured in six-pore plates, incubated at 37 °C with 5% CO_2_ for 18–24 h. Three hours before transfection, cells at about 80–90% confluency were changed to the serum and antibiotic-free media. Then, cells were transfected using lipofectamin 2000 reagent (Life Technologies, Gaithersburg, MD, USA) referring to manufacturer’s instructions and incubated at the same conditions as above for 48 h. In addition to the in vivo experiment, based on the manual, concentrated lentiviral solutions of sh-ZFAS1 was mixed with two wells of nutrient solution contains HONE-1 cell, respectively. Finally, the cells were digested by pancreatin and injected into mice after incubation for 48 h.

### Isolation of cytoplasmic and nuclear RNA

Follow the manufacturer's instructions to isolate and purify cytoplasmic and nuclear RNA using the cytoplasmic and nuclear RNA purification kit (Sigma-Aldrich). U6 is regarded as an internal reference for nuclear RNA.

### RNA isolation and qRT-PCR

The RNA in the two cell lines was extracted with TRIzol reagent (Invitrogen) following the instruction of manufacturer. The concentration and purity of extracted RNA were detected by a NanoDrop Spectrophotometer (Thermo Scientific, Waltham, MA, USA). Then cDNA was synthesized using the Reverse Transcription System Kit (Applied Biosystems, Foster City, CA, USA). The detection of ZFAS1, GAPDH and miR-100-3p expression was performed by RT-PCR using SYBR Green PCR Master Mix (Takara, Dalian, China) and TaqMan MicroRNA Assay Kit (Applied Biosystems) on Bio-Rad iQ5 Multicolor Real-Time qRT-PCR Detection system (Bio-Rad, Hercules, CA, USA). GAPDH was a reference gene for ZFAS1 and METTL3, while U6 was the reference gene for miR-100-3p. The primers were synthesized by Sangon (Additional file 5: Table S2) (Shanghai, China). All statistics were analyzed based on 2^−ΔΔCt^ method.

### m6A RNA methylation quantification

According to the manufacturer's instructions, the EpiQuik m6A Methylation Quantitative Kit (colorimetric; Epigentek) measured the total m6A level of the extracted RNA. Poly-apurified RNA (200 ng) was used for each sample analysis.

### RNA immunoprecipitation (RIP)

RIP was performed using the EZ-Magna RIP kit (Millipore) according to the instructions. Briefly, 24 h after transfection, cells were collected and RIP assay was performed using m6A antibody (2 mg/sample; Synaptic system) or AGO2 antibody (5 mg/ sample; Abcam). IgG was used as a negative control. As described earlier, quantitative RT-PCR was used to detect the co-precipitated RNA. In the analysis of mature RNA, comparative CT (DDCT) methods were used. The input fractional CT values were used to normalize the differences in sample preparation, and negative control (IgG) CT was used to adjust the background scores.

### CCK-8 and Wound-healing assays

For CCK-8 detection, cells were seeded into 96-well plates at the density of 1000 cells per well, and 10 mL of CCK-8 (Dojindo) was added to each well on days 0–5. Then incubated with the cells in 37℃ 2 h, the density of light measured at 450 nm.

In the wound healing assays, the cells were inoculated in a 6-well plate and cultured to the fusion state. After starvation in serum-free medium for 24 h, monolayers were linearly scraped to introduce artificial wounds and captured at 0 h and 24/48 h.

### Luciferase reporter assays

293 T cells were transferred to 24-well plates in each well and incubated before transfection. Then the cells were co-transfected using miR-100-3p, the wild type or mutant ZFAS1 and ATG10. After 48 h of transfection, the cells were washed by PBS before incubated with shake for 15 min at room temperature. Then cell lysis buffer was collected at 4 °C and centrifuged for 2 min. The supernatant was collected and refrigerated at -80 °C refrigerator. Cell lysis buffer was added to 96-well plate with 10 μl per well. During the detection, 30 μl luciferase reagents II (Promega, Madison, WI, USA) were added. Then 30 μl stop buffer was added to terminate the activities. The luciferase activities were detected by the Dual-Glo luciferase reporter assay kit (Promega) for 48 h post transfection.

### Flow cytometric analysis of apoptosis

After transfection, 2 × 10^5^ cells from the transfection group and the NC group were collected, washed and resuspended with precooled PBS according to the Annexin V Apoptosis Detection Kit (Life Technologies). Then the cells were incubated for 15 min before washed and resuspended in 500 μl of binding buffer. Ten microliter of Annexin-V–FITC and propidium iodide respectively were added and the mixture was analyzed using the FACS Calibur. The data was analyzed by FACS Diva software.

### In vivo nude mouse models

All of the experimental procedures involving animals were approved by the Institutional Animal Care and Use Committee of the Sichuan University. Female BALB/c nude mice (ages 4–5 weeks, 18–20 g) were purchased from the Charles River Laboratories. For the tumor growth model, 1 × 10^6^ HONE-1 sh-NC or sh-ZFAS1 cells were injected into the axilla of the mice, and the tumor size was measured every 3 days (Fig. 8A). On day 30, the mice were killed, and the tumors were dissected and weighed.

### Immunohistochemistry

Immunohistochemical staining was done on formalin-fixed and paraffin embedded tissue sections from tumors tissue. The tissue was placed in the incubator at 65 °C for 2 h before removing paraffin by xylene. After gradient elute the tissue by absolute ethyl alcohol, 95% ethyl alcohol, 85% ethyl alcohol, washed the tissue by redistilled water. In the third step, target retrieval was achieved with BOND Novocastra Epitope Retrieval Solution 1 (Leica Biosystems) at 100 °C for 20 min. Monoclonal Anti-ki67 antibody (2 µg/ml, Abcam) were used as primary antibody. After that, secondary antibody horseradish peroxidase-conjugated goat anti-rabbit IgG (1:5000, Abcam) were used. In this way, ki67 was stained in the tissue and the tissue was photographed by optical microscope.

### Immunofluorescence assay

The cells were fixed and blocked with 3% bovine serum albumin, and then incubated with rabbit anti-LC3II (ab48394, 1:1000, USA) at 4 °C overnight. Then it was incubated with a red fluorescent secondary antibody (Intertek) for 2 h, stained with DAPI (1 μg/mL) for 5 min, sealed, and observed and photographed under a fluorescence microscope (Olympus Optical Co., Ltd).

### Hematoxylin–eosin (HE) staining

The 5-μm tissue sections were deparaffinized in xylenol for 20 min, rehydrated with reduced ethanol concentrations (100%, 90%, 70%) for 5 min, and then washed with water. For HE staining, the slides were stained in Mayer's Hematoxylin Solution for 5 min and then counterstained with Eosin for 5–10 min.

### RNA stability assays

HON3-1 cells with or without METL3 knockout were treated with Actinomycin D at a final concentration of 5 μg/ml for 0, 2 and 4 h, and then collected. Total RNA was separated with TRIzol (Invitrogen) and analyzed by RT-PCR.

### Western blot (WB)

First of all, proteins were isolated from extraction reagent (Thermo Fisher Scientific), followed with separated and transferred some proteins to Immobilon™-P membranes (Merck Millipore, Billerica, USA). Secondly, incubating ATG10 (ab124711,1:5000), LC3 (ab192890, 1:2000), P62 (ab109012, 1:10,000), Beclin1 (ab210498, 1:1000), p-PI3K (ab182651, 1:1000), PI3K (ab32089, 1:1000), p-AKT (ab38449, 1:1000), AKT (ab179463, 1:10,000), p-mTOR (ab109268, 1:5000), mTOR (ab134903, 1:10,000), E-cadherin (ab76055, 1:500), N-cadherin (ab76011, 1:10,000), vimentin (ab92547, 1:2000) and GAPDH (ab8245, 1:5000) (Abcam) primary antibodies against mouse in the membrane at 4 °C for a night time. Washed with TBST after incubation immediately, hybridized membranes with horseradish peroxidase (HRP)-linked antibody goat anti-rabbit IgG (1:2000, Abcam) for 1 h. At least, checking the antibody binding by an enhanced chemiluminescence kit.

### Statistical analysis

SPSS 22.0 software (SPSS Software, USA) was used for statistical analysis and data was expressed with mean ± standard deviation. Data were compared by unpaired t-test (differences between two groups) or one-way ANOVA analysis (differences among groups). * *p* value < 0.05 was regarded statistically significant.

## Result

### ZFAS1 upregulation in NPC and its association with poor clinical outcome

**Fig. 1:**
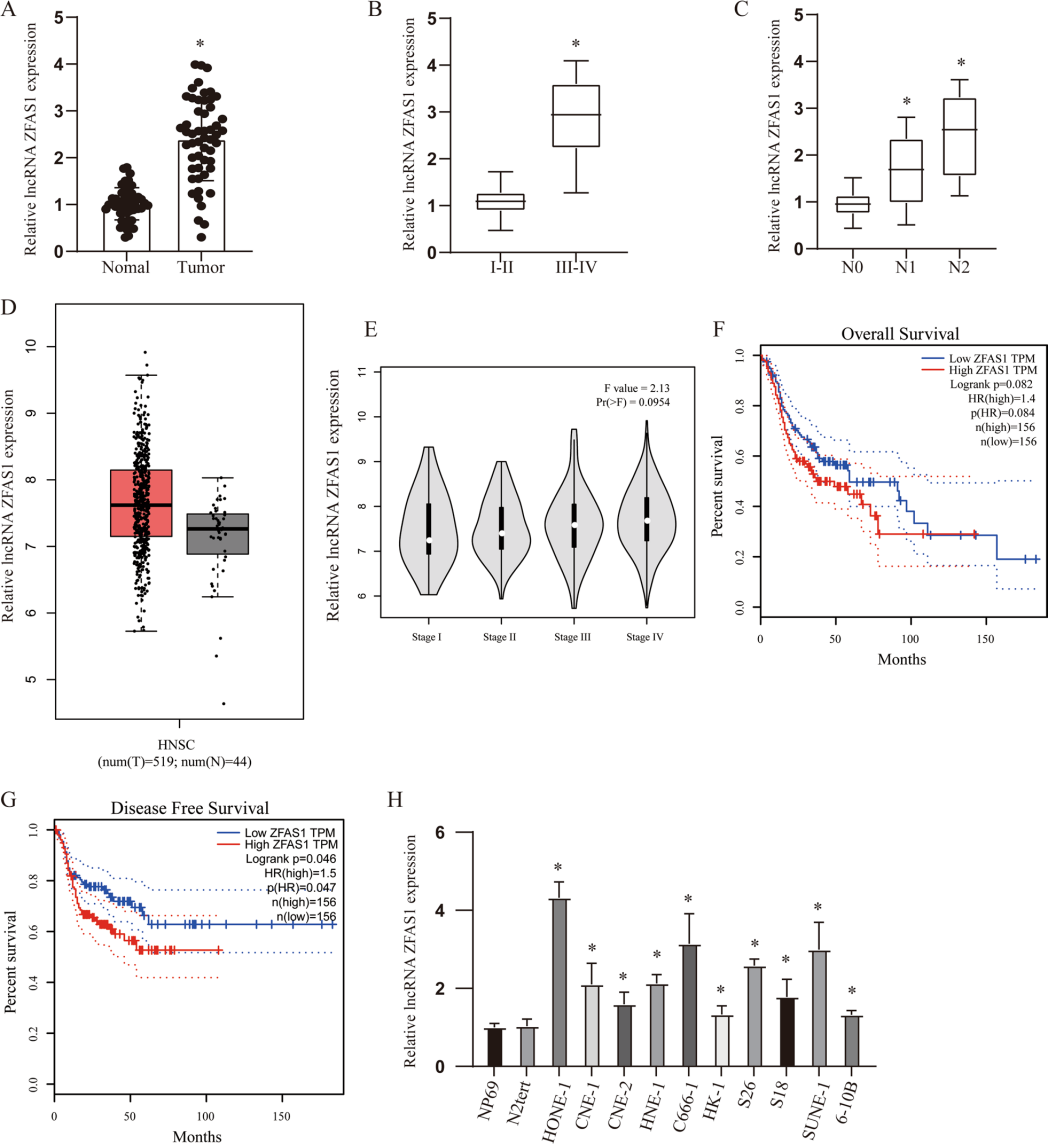
ZFAS1 upregulation in NPC and its association with poor clinical outcome. **A** The expression of ZFAS1 in 53 pairs of patient samples. **B**, **C** The expression level of ZFAS1 was positively correlated with tumor stage (**B**) and lymph node metastasis (**C**). **D** The expression level of ZFAS1 in the TCGA database. **E** The expression of ZFAS1 in different tumor tissue stages in TCGA database. **F** Kaplan–Meier survival analysis of ZFAS1 expression for overall survival. **G** Kaplan–Meier survival analysis of ZFAS1 expression for disease free survival (DFS). **H** ZFAS1 expression level in the immortalized NP69 and N2Tert cells and 10 NPC cell lines. *p < 0.05, compared with relative control group

First, we detected the expression of ZFAS1 in tumor tissues and adjacent tissues of 53 NPC patients and found that it was highly expressed in tumor tissues (Fig. 1A, p  < 0.05). Further analysis revealed that the expression level of ZFAS1 gradually increased with late stage and lymph node metastasis (Fig. 1B, C, p < 0.05). In addition, the analysis of NPC samples in TCGA data through the GEPIA database also found this phenomenon of increased expression. However, the difference in tumor staging was not statistically significant (Fig. 1D, E). Furthermore, Kaplan–Meier survival analysis revealed that patients with high ZFAS1 expression had worse overall and disease-free survivals (Fig. 1F, G).

After extended analysis of TCGA data, it was found that ZFAS1 expression was up-regulated in a variety of tumors (Additional file 1: Figure S1A). And it has been shown to have a significant correlation with poor prognosis in a variety of tumors, such as low grade glioma (LGG), liver hepatocellular carcinoma (LIHC), Adenoid Cystic Carcinoma (ACC) and Ovarian serous cystadenocarcinoma (OV) (Additional file 1: Fig. 1B–E, all *p* < 0.05). These results indicate that ZFAS1 has been confirmed in a variety of tumors as an oncogene. Similarly, ZFAS1 expression was significantly higher in 10 NPC cell lines than that in control cells (Fig. 1H, p < 0.05).

### Effects of ZFAS1 on the proliferation and apoptosis of NPC cells

**Fig. 2:**
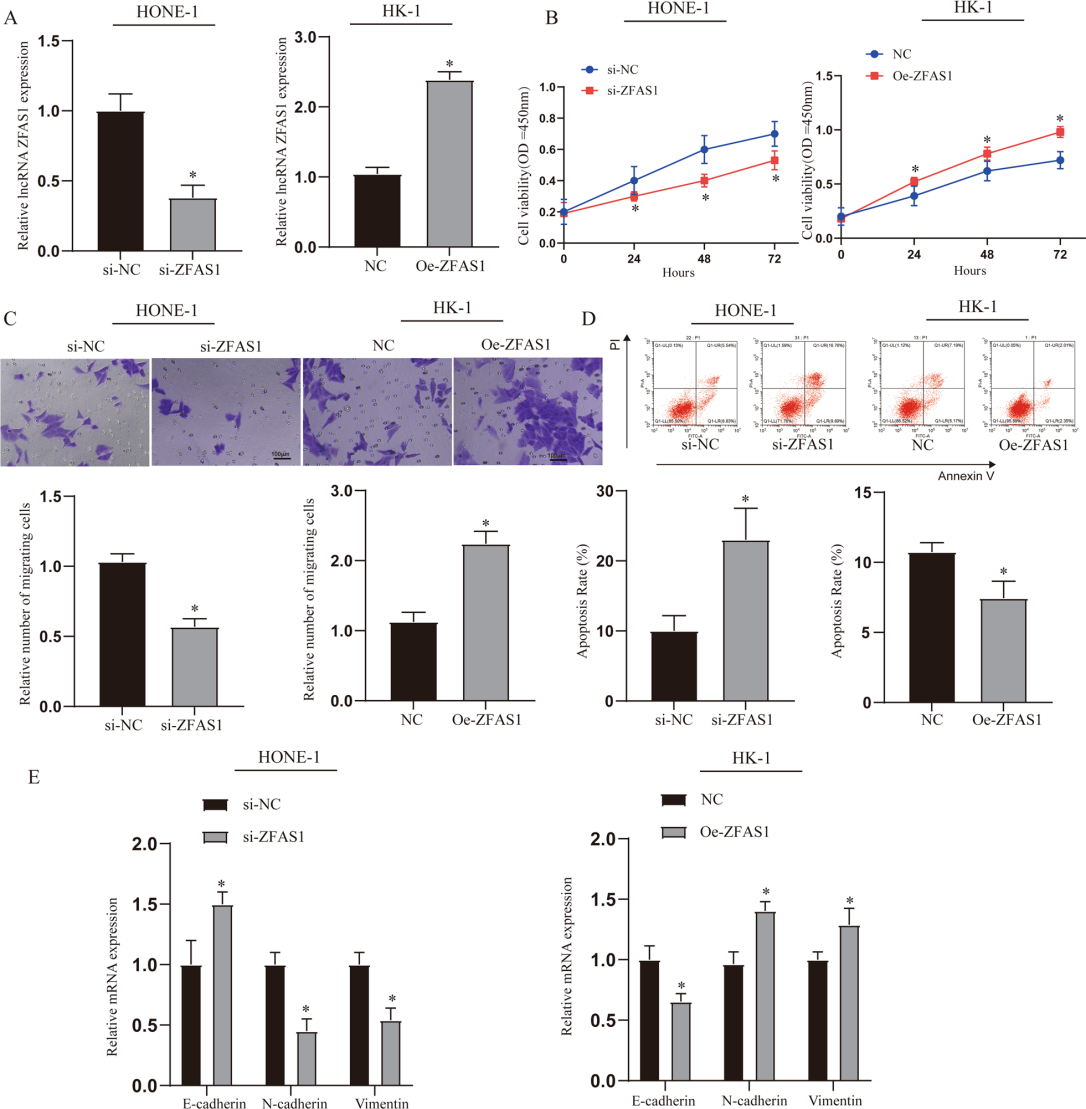
Effects of ZFAS1 on the proliferation and apoptosis of NPC cells. **A** Detect the expression of ZFAS1 after transfecting si-ZFAS1 and oe-ZFAS1 into HONE-1 and HK-1 cells. **B** CCK8 detects the cell viability of each treatment group. **C** Transwell detected the migration of NPC in each treatment group. **D** Detection of NPC cell apoptosis in different treatment groups. **E** Expression of EMT related genes and proteins. *p < 0.05, compared with NC or si-NC group. NC: normal control, oe-ZFAS1: over-expression ZFAS1, si-NC: Small interfering RNA NC, si-ZFAS1: Small interfering RNA ZFAS1

Through the above experiments, we found that the expression of ZFAS1 was up-regulated in tumor tissues and NPC cells. In order to verify the effect of ZFAS1 on NPC cells, we constructed an intervention model of ZFAS1 expression. The expression of ZFAS1 was detected by qRT-PCR to verify that the suppression and overexpression models were successfully constructed (Fig. 2A, p < 0.05). The CCK8 assay results showed that compared with the NC group, inhibiting the expression of ZFAS1 could inhibit the viability of NPC cells, while overexpression of ZFAS1 was the opposite (Fig. 2B, p < 0.05). This phenomenon was also found when the cell migration ability was tested by transwell (Fig. 2C, p < 0.05). In the detection of cell apoptosis, we found that inhibiting the expression of ZFAS1 in HONE-1 cell could significantly promote the increase in the rate of apoptosis, while overexpression of ZFAS1 in HK-1 cell could significantly inhibit the occurrence of apoptosis. (Fig. 2D, p < 0.05). Finally, we found through the detection of epithelial-mesenchymal transition (EMT)-related genes that interfering with the expression of ZFAS1 can significantly affect the EMT process, which may be one of the reasons for its cancer-promoting effect (Fig. 2E, p < 0.05).

### The m6A modification improves the stability of ZFAS1 and promotes its expression

**Fig. 3:**
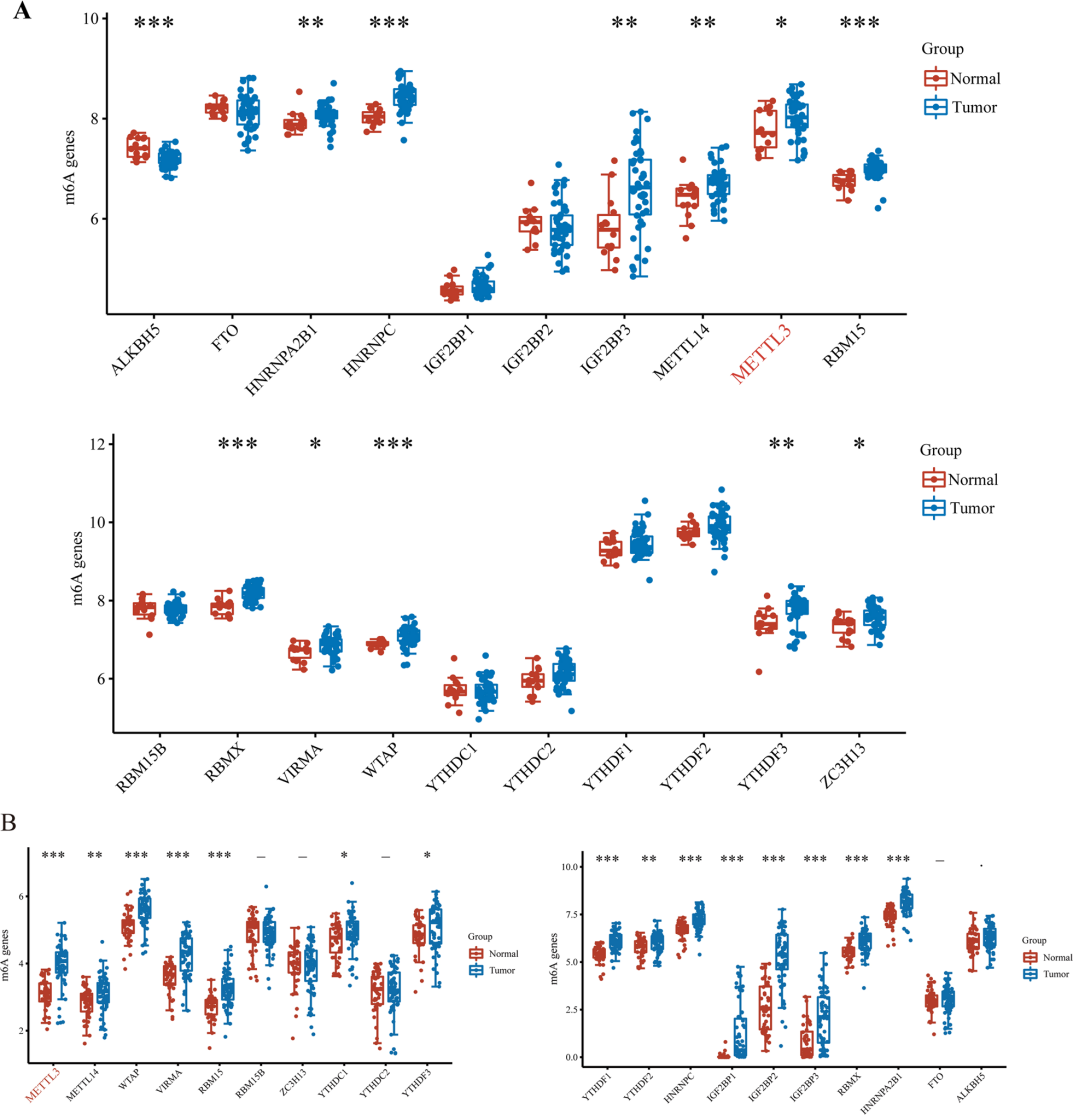
GEO and TCGA database analysis of m6A-related genes in NPC patients. **A** M6A related gene analysis results. The expression distribution of m6A mRNA in tumor and normal groups, where the horizontal axis represents different mRNA, the vertical axis represents the mRNA expression distribution, where different colors represent different groups, and the upper left corner represents the significance *p* value test method. **B** The expression distribution of m6A mRNA in tumor tissues and normal tissues, where the horizontal axis represents different mRNA, the vertical axis represents the mRNA expression distribution, where different colors represent different groups, and the upper left corner represents the significance *p* value test method. Asterisks represent levels of significance *p < 0.05, **p < 0.01, ***p < 0.001

Recently, frontier research has shown that m6A modifications in mRNA and lncRNA are extremely extensive, and functionally regulate eukaryotic transcriptome to affect RNA splicing, export, localization, translation and stability [15, 16]. Similarly to previous studies [17], we obtained GSE12452 and GSE64634 NPC analysis chip data from the GEO database (N = 14, T = 43). First, we standardized the acquired data (Additional file 2: Fig. 2A–C), and then compared the NPC samples with normal tissues and found that there were a large number of differentially expressed m6A-modified related genes in NPC, such as ALKBH5 as m6A erasers (*p* < 0.001), YTHDF3 as m6A readers (*p* < 0.01), and m6A as m6A METTL3 of writers (*p* < 0.05) (Fig. 3A). Furthermore, we analyzed the data from the TCGA database (excluding the data of patients treated with radiotherapy and chemotherapy) and found that a large number of m6A-modified genes are differentially expressed (Fig. 3B). These results indicate that m6A modification may be one of the important mechanisms for the development of NPC. In addition, METTL3 was significantly upregulated in both the above GEO and TCGA analyses, which is consistent with our hypothesis.

### METTL3 regulates the expression of ZFAS1

**Fig. 4:**
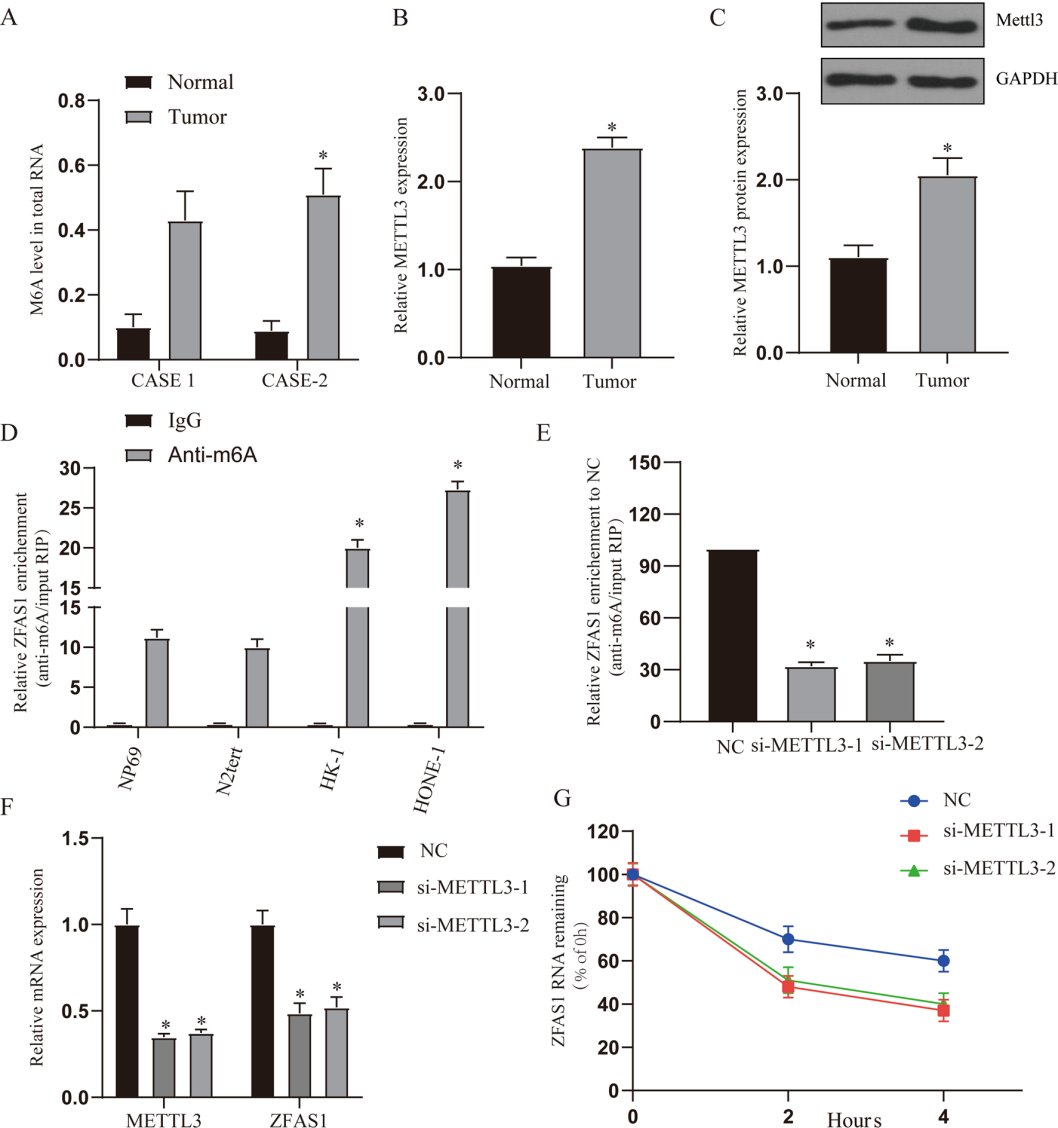
METTL3 regulates the expression of ZFAS1. **A** The level of m6A modification in total RNA in clinical samples (case1 and case2), n = 3. **B**, **C** QRT-PCR and WB detect the expression of METTL3 mRNA and protein, n = 53. **D** The M6A methylation level of ZFAS1 in normal nasopharyngeal epithelial cells (NP69 and N2Tert) and NPC cells (HK-1 and HONE-1) was determined by MeRIP-qPCR analysis. **E** The change of m6A-modified ZFAS1 increased when METTL3 interfered with the expression. The input RNA fraction Ct value was used to account for RNA sample preparation differences, and negative control groups (IgG) were used to adjust background fraction. **F** The graph shows METTL3 and ZFAS1 expression upon METTL3 knock down. **G** Compared with the control, the stability of ZFAS1 RNA was reduced in HHONE-1 cells with knockout of METL3 gene. Treat the cells with 5 mg/mL Actinomycin D, and isolate RNA at 0, 2, and 4 h. *p < 0.05, compared with relative control group. NC: normal control, si-METLL31: Small interfering RNA METTL3-1, si-METLL32: Small interfering RNA METTL3-2

Based on the above results, we hypothesized that METTL3 may be involved in the regulation of ZFAS1 expression. In order to further verify the role of m6A modification in NPC, we first detected the level of m6A modification in patient samples and found that the level of m6A modification in tumor tissues was significantly higher than that in adjacent tissues (Fig. 4A, p < 0.05). Further detection of METTL3 expression showed that the expression of METTL3 in tumor tissues was significantly higher than that in adjacent tissues (Fig. 4B, C, p < 0.05). The results in the TCGA database were the same as those in the clinical samples (Additional file 2: Fig. 2D, E). In addition, correlation analysis showed a positive correlation with ZFAS1 expression (R = 0.13, P = 0.002) (Additional file 2: Fig. 2F).

Next, we performed methylated RNA immunoprecipitation (Me-RIP) analysis in 2 nasopharyngeal epithelial cells (NP69 and N2Tert) and 2 NPC cells (HK-1 and HONE-1). The results showed that m6A levels were higher in HONE-1 and HK-1 cells than in NP69 and N2Tert cells (Fig. 4D, p < 0.05). We performed siRNA-mediated silencing of METLL3, the core component of the m6A methylase complex, and found that the down-regulation of METLL3 resulted in a decrease in the levels of m6A total RNA and ZFAS1 RNA (Fig. 4E, p < 0.05). Then we investigated whether m6A modification affects the RNA metabolism of ZFAS1. Knockout of METTL3 can reduce the total expression of ZFAS1 (Fig. 4F, p < 0.05). Then we measured the loss of ZFAS1 RNA after blocking the synthesis of new RNA with actinomycin D. The results showed that the RNA stability of ZFAS1 decreased after METTL3 was silenced (Fig. 4G, p < 0.05). These findings increase the possibility that ZFAS1 has a higher m6A level in NPC, and ZFAS1 modified by m6A improves its transcriptional stability, which may be part of the reason for the significant upregulation of ZFAS1 in NPC.

### ZFAS1 exerts effect by competitively binding miR-100-3p

**Fig. 5:**
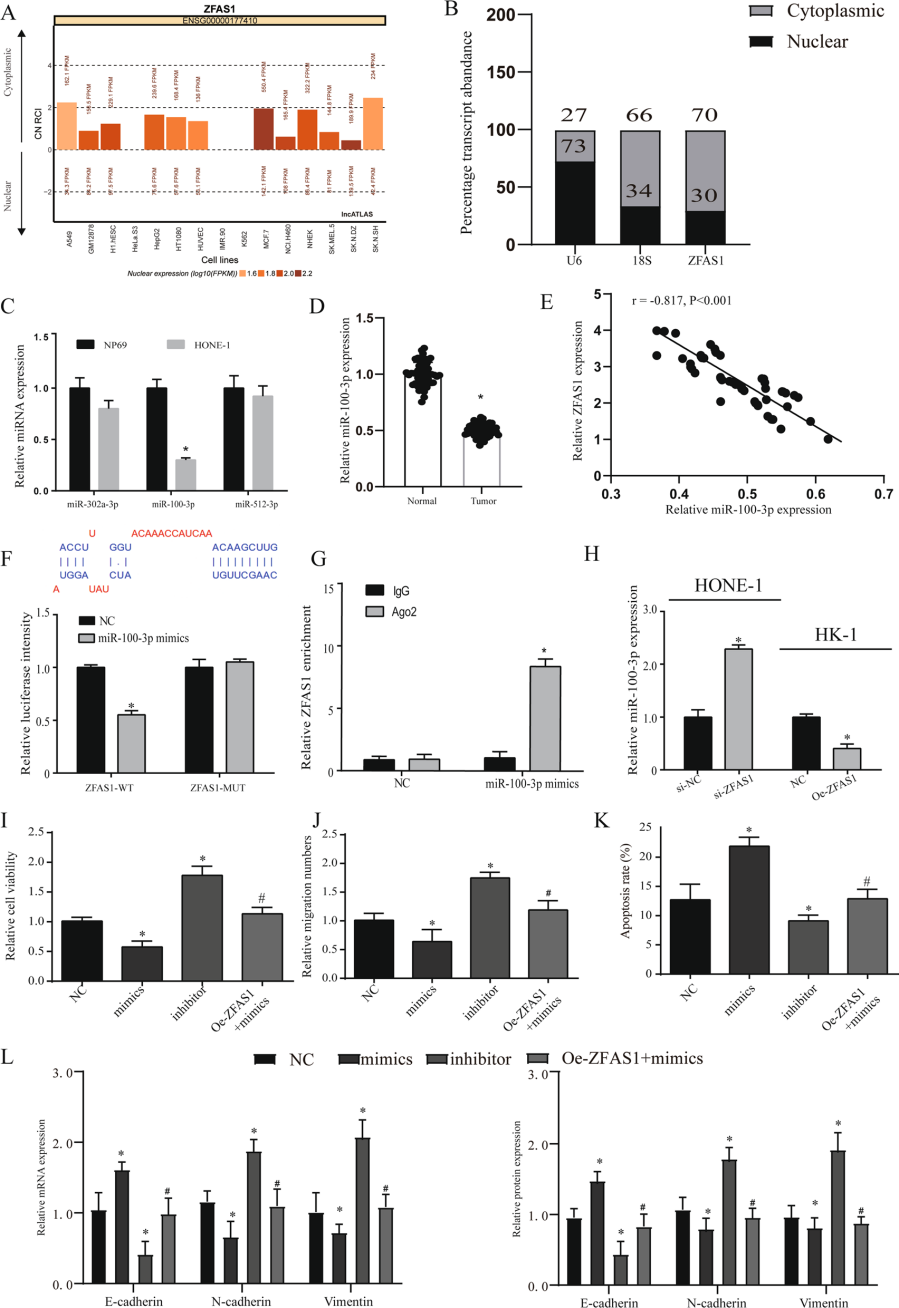
ZFAS1 exerts a cancer-promoting effect by targeting miR-100-3p. **A** Use the bioinformatics tools in lncATLAS to predict the subcellular localization of ZFAS1. **B** QRT-PCR analysis of subcellular ZFAS1 expression in the nucleus and cytoplasm of HONE-1 cells. U6 and 18S were used as endogenous controls. **C** QRT-PCR was used to detect the expression levels of miR-100-3p, miR-302a-3p and miR-512-3p in HONE-1 and HK-1 cells. **D** The expression of miR-100-3p in clinical samples, n = 53. **E** Pearson correlation was used to detect the correlation between the expression of miR-100-3p and ZFAS1. **F** Dual luciferase report verifies the targeting of ZFAS1 and miR-100-3p. The binding site prediction comes from the lncbase database. **G** RIP assay verifies the targeting of ZFAS1 and miR-100-3p. **H** The expression of miR-100-3p in different treatment groups in HONE-1 and HK-1 cells. **I** CCK8 detects the cell viability of each treatment group. **J** Transwell detected the migration of NPC in each treatment group. **K** Detection of NPC cell apoptosis in different treatment groups. **L** Expression of EMT related genes and proteins. *p < 0.05, compared with NC group, #*p* < 0.05, compared with mimics group. NC: normal control, oe-ZFAS1: over-expression ZFAS1, si-NC: Small interfering RNA NC, si-ZFAS1: Small interfering RNA ZFAS1

In order to explore how ZFAS1 exerts its function, we used lncATLAS (http://lncatlas.crg.eu/) to predict its subcellular location. ZFAS1 is expected to be mainly located in the cytoplasm of all available cell types (Fig. 5A). QRT-PCR analysis of ZFAS1 in the nucleus and cytoplasm showed that ZFAS1 was mainly located in the cytoplasm of nasopharyngeal carcinoma cells (Fig. 5B, p < 0.05). Based on the above results, we speculate that ZFAS1 functions through a ceRNA mechanism. Furthermore, we performed microarray analysis on tumor tissues and adjacent tissues to screen differentially expressed miRNAs, and the results showed up-regulated and down-regulated top10 miRNAs (Additional file 3: Fig. 3A). The target genes of ZFAS1 and the difference results were screened out by starbase database for miR-100-3p, miR-512-3p and miR-302a-3p for further analysis (Additional file 3: Fig. 3B). The expression of the three miRNAs was detected by HONE-1 cells, only the reduction of miR-100-3p was found to be significantly different (Fig. 5C, p < 0.05). After analyzing the clinical samples and TCGA, we found that miR-100-3p was down-regulated in tumor tissues (Fig. 5D, Additional file 3: Fig. 3C, all *p* < 0.05). Through correlation analysis, it is found that ZFAS1 and miR-100-3p are negative correlated (r = -0.817, *p* < 0.001) (Fig. 5E).

Finally, we used the starbase 3.0 database to predict their possible binding sites, and through the dual luciferase reporter assay and RIP assay, we proved that miR-100-3p was the target gene of ZFAS1 (Fig. 5F, G, p < 0.05). Furthermore, we used HK-1 and HONE-1 cells to detect that interference with ZFAS1 can effectively regulate the expression of miR-100-3p (Fig. 5H, p < 0.05).

### ZFAS1 exerts a cancer-promoting effect by targeting miR-100-3p

First, we tested the expression of miR-100-3p in each cell model and found that mimics can overexpress miR-100-3p, and inhibitors can significantly inhibit the expression of miR-100-3p. However, when ZFAS1 and mimics were transfected at the same time, the upregulation of miR-100-3p was reversed (Additional file 3: Fig. 3D, p < 0.05). In order to verify whether ZFAS1 exerts a cancer-promoting effect through miR-100-3p, we tested that overexpression of miR-100-3p can reduce the viability and migration ability of NPC cells, and this phenomenon was reversed after overexpression of ZFAS1 (Fig. 5I, J, all *p* < 0.05). We further found that ZFAS1 can regulate NPC cell apoptosis through miR-100-3p (Fig. 5K, p < 0.05). We also found similar phenomena in the related genetic testing of the EMT process. These results suggest that ZFAS1 can promote cancer through sponge miR-100-3p (Fig. 5L, p < 0.05).

### MiR-100-3p regulates autophagy levels by targeting ATG10

**Fig. 6:**
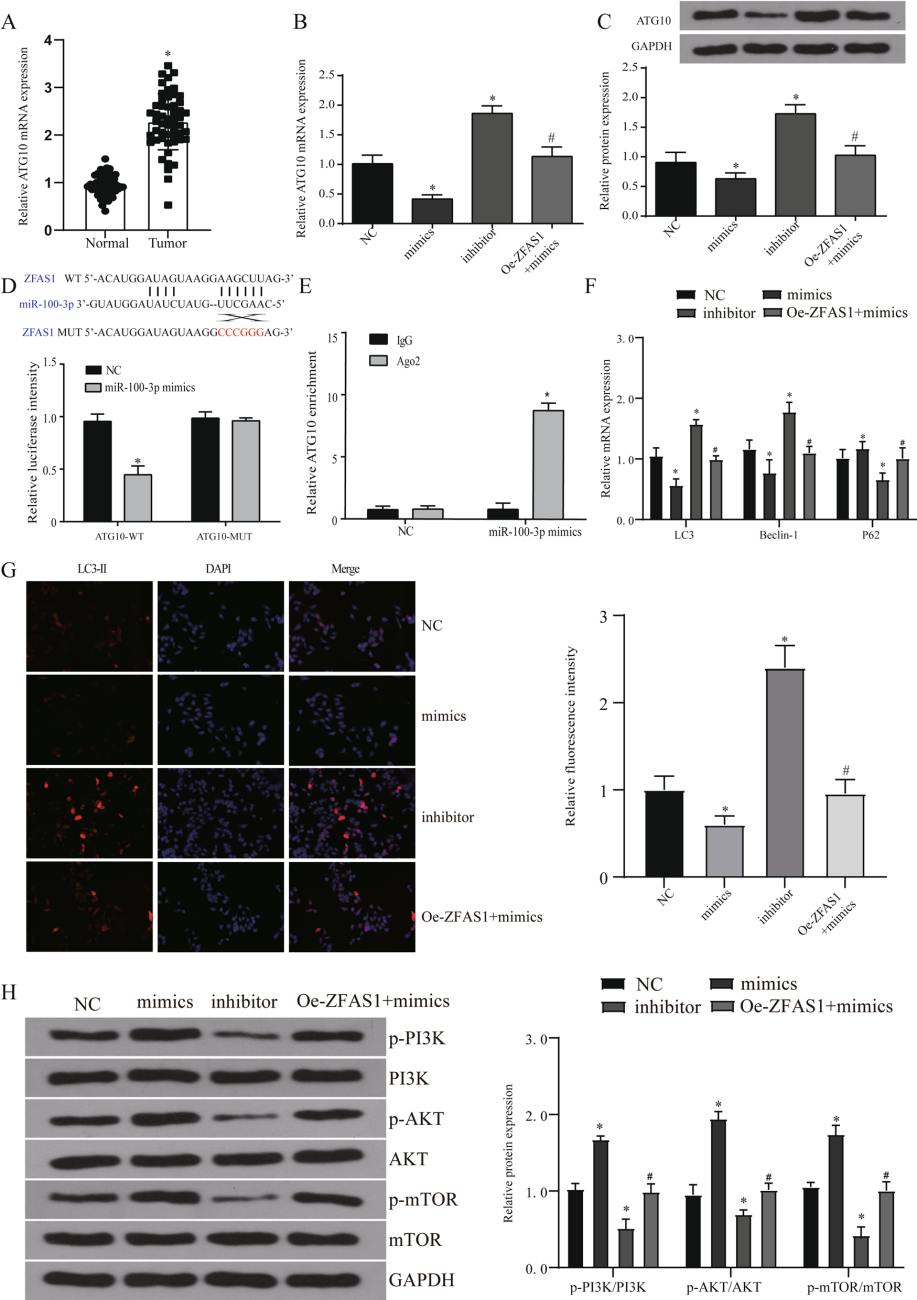
ZFAS1 affects the level of autophagy by regulating the expression of miR-100-3p. **A** The expression of ATG10 in 53 pairs of patient samples. **B**, **C** The expression of ATG10 gene (**B**) and protein (**C**) in each treatment group. **D** Dual luciferase report verifies the targeting of ATG10 and miR-100-3p. The binding site prediction comes from the starbase database. **E** RIP assay verifies the targeting of ATG10 and miR-100-3p. **F** QRT-PCR detects the expression of autophagy-related genes (LC3, Beclin1, p62) in different treatment groups. **G** Immunofluorescence was used to detect the expression of LC3II label in each treatment group. **H** WB detected the expression of PI3K/AKT signaling pathway in different treatment groups. *p < 0.05, compared with NC group, #*p* < 0.05, compared with mimics group. NC: normal control, oe-ZFAS1: over-expression ZFAS1

In order to further explore the role of miR-100-3p in tumors, we detected the target genes of miR-100-3p through miRwalk and targetscan and found that a total of 941 target genes were enriched (Additional file 4: Fig. 4A). GO analysis of target genes using DAVID database showed that autophagy plays an important role in it (Additional file 4: Fig. 4B). We screened the genes ATG10 and ATG5 related to autophagy enrichment for further testing and found that only ATG10 was significantly up-regulated in NPC cells and tumor tissues (Additional file 4: Fig. 4C, D, Fig. 6A, all *p* < 0.05). In the TCGA database analysis, we verified the high expression of ATG10 and its negative correlation with miR-100-3p. The expression of ATG10 and ZFAS1 are positively correlated in HNSC (Additional file 4: Fig. 4E–G).

We transfected miR-100-3p mimics, inhibitor and oe-ZFAS1 + mimics into HONE-1 cells. Firstly, the expression of ATG10 in each treatment group was detected by qRT-PCR and WB. The results show that miR-100-3p can effectively regulate the expression of ATG10 and this regulation is affected by ZFAS1 (Fig. 6B, C, all *p* < 0.05). The detection of luciferase reporter assays showed that in NPC cells transfected with wild-type ATG10 3’UTR reporter gene plasmid, overexpression of miR-100-3p can significantly inhibit the luciferase activity, while those transfected with mutant reporter gene plasmid In the cells, luciferase activity was not significantly inhibited (Fig. 6D). Because Ago2 is a core component of the RISC that participates in miRNA-mediating mRNA destabilization or translational repression, we conducted RIP assays using an anti-Ago2 antibody, which showed that endogenous ZFAS1 was preferentially enriched in Ago2-RIPs compared with control IgG-RIPs (Fig. 6E). These results indicate that miR-100-3p can play a role by targeting the expression of ATG10.

We detected autophagy-related genes (LC3, Beclin1, P62) and found that miR-100-3p can inhibit autophagy, and this regulatory effect can be reversed by ZFAS1 (Fig. 6F, all *p* < 0.05). The detection of LC3 labeling by immunofluorescence also verified this conclusion (Fig. 6G). These results indicate that ZFAS1 can regulate the change of autophagy level through miR-100-3p/ATG10 pathway.

### The role of PI3K/AKT signaling pathway in the regulation of autophagy by ZFAS1

**Fig. 7:**
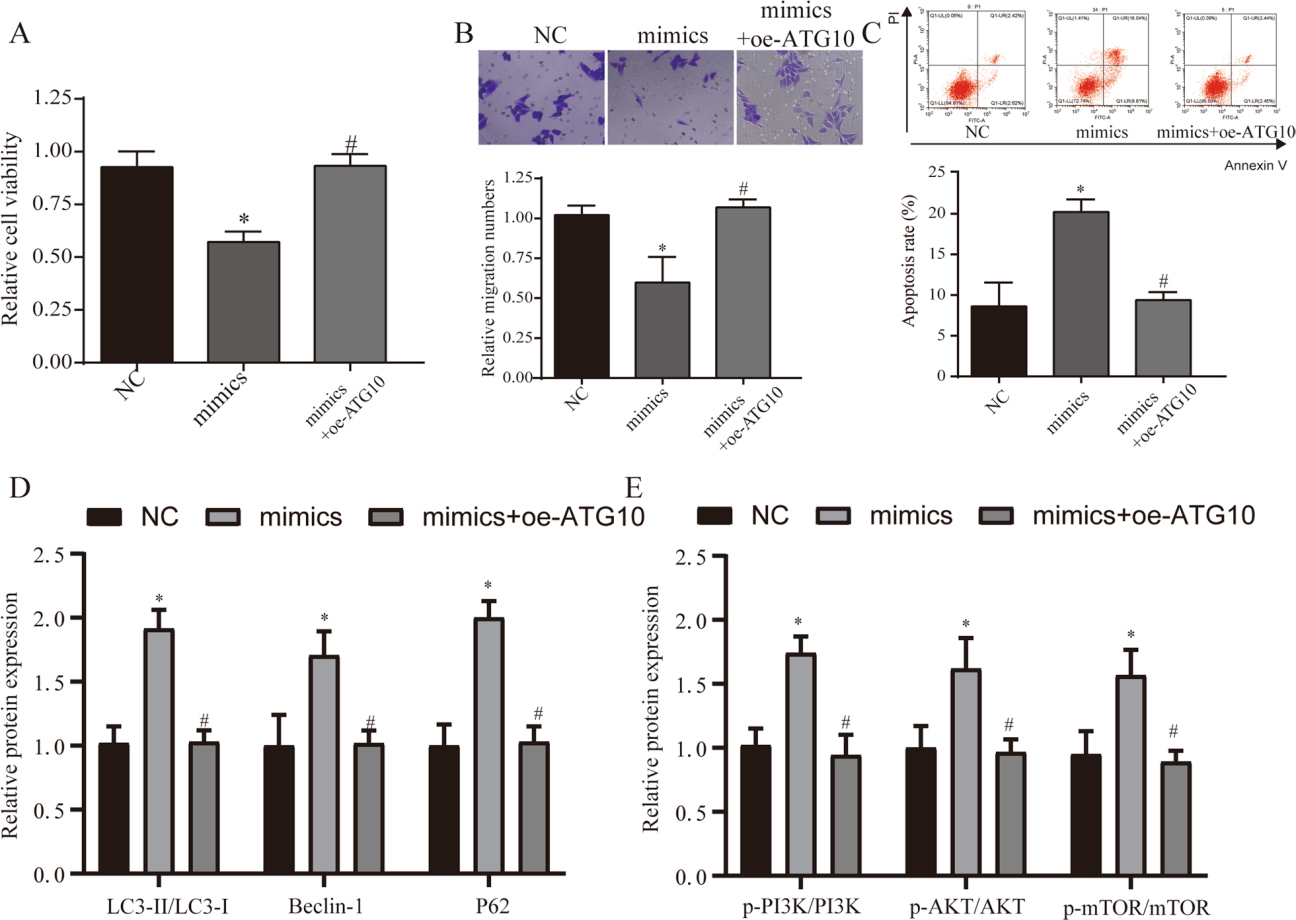
MiR-100-3p affects the level of autophagy by regulating the expression of ATG10. **A** CCK8 detects the cell viability of each treatment group. **B** Transwell detected the migration of NPC in each treatment group. **C** Detection of NPC cell apoptosis in different treatment groups. **D** WB detects the expression of autophagy-related genes (LC3, Beclin1, p62) in different treatment groups. **E** WB detected the expression of PI3K/AKT signaling pathway in different treatment groups. *p < 0.05, compared with NC group, #*p* < 0.05, compared with mimics group. NC: normal control, oe-ATG10: over-expression ATG10

A large number of studies have shown that the PI3K/AKT pathway is one of the important pathways in the regulation of autophagy [18], so we further verify whether ZFAS1 plays a regulatory role through the PI3K/AKT pathway. We transfected miR-100-3p mimics, inhibitor, oe-ZFAS1 + mimics and oe-ATG10 + mimics into HONE-1 cells. We found through WB detection that miR-100-3p can significantly inhibit the PI3K/AKT pathway, and this inhibition was reversed by the overexpression of ZFAS1 (Fig. 6H). In the detection of NPC cell viability, migration and apoptosis ability, it was found that overexpression of ATG10 can effectively interfere with the tumor cell process regulated by miR-100-3p (Fig. 7A–C). After further testing the autophagy markers and PI3K/AKT signaling pathway, it was found that the overexpression of ATG10 can reverse the regulation of miR-100-3p on autophagy and signaling pathways (Fig. 7D, E). The above results indicate that ZFAS1 can regulate the expression of ATG10 through sponge miR-100-3p to affect the level of autophagy, and the pathway that plays this role may be the PI3K/AKT pathway.

### In vivo experiments prove that ZFAS1 can regulate tumor and autophagy

**Fig. 8:**
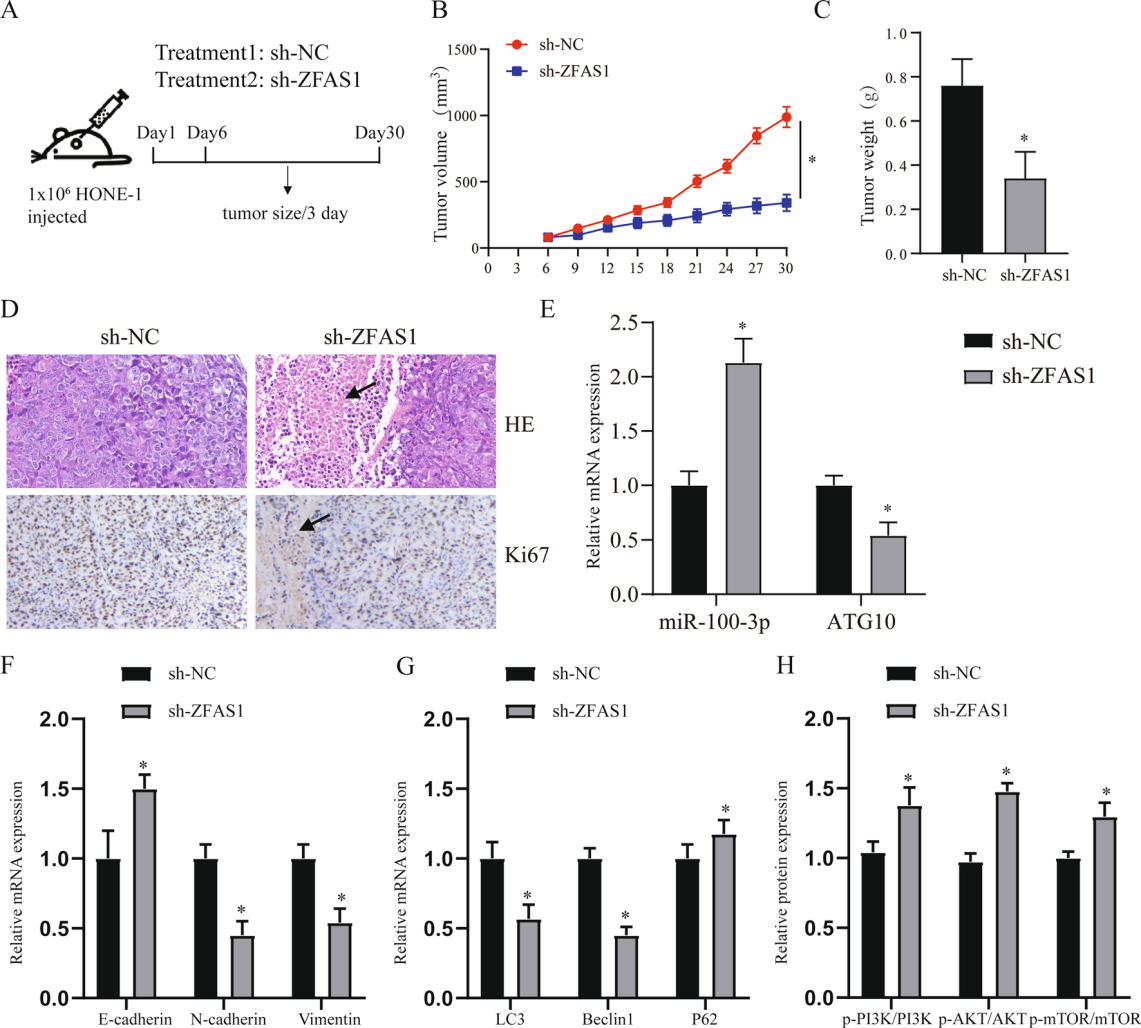
In vivo experiments prove that ZFAS1 can regulate tumor and autophagy. **A** HONE-1 cells stably expressing sh-ZFAS1 or hybrid control were transplanted into the axilla of nude mice to establish a tumor growth model. Representative image of the formed tumor (**A**), tumor volume growth curve (**B**) and weight (**C**). **D** HE and Ki67 staining in the tumors of each treatment group. The arrow points to the area of tumor necrosis. **E** After transfection of sh-ZFAS1, qRT-PCR was used to detect the expression of miR-100-3p and ATG10. **F** Expression of EMT related genes. **G** QRT-PCR detects the expression of autophagy-related genes (LC3, Beclin1, p62) in different treatment groups. **H** WB detected the expression of PI3K/AKT signaling pathway in different treatment groups. *p < 0.05, compared with sh-NC group. Sh-NC: short hairpin RNA contrl, sh-ZFAS1: short hairpin RNA ZFAS1

Finally, we confirmed the ability of ZFAS1 to promote NPC proliferation and autophagy in vivo. The tumor growth model showed that ZFAS1 knockdown significantly inhibited tumor growth (Fig. 8A–C, p < 0.05). Similarly, the results of HE and Ki67 staining confirmed that sh-ZFAS1 can inhibit tumor growth (Fig. 8D). QRT-PCR result shown that sh-ZFAS1 could promote miR-100-3p and inhibit the expression of ATG10 (Fig. 8E, p < 0.05). We found through the detection of epithelial-mesenchymal transition (EMT)-related genes that interfering with the expression of ZFAS1 can significantly affect the EMT process (Fig. 8F, p < 0.05). Finally, we detected autophagy markers and PI3K/AKT pathway related genes and found that ZFAS1 can affect the level of autophagy by affecting the PI3K/AKT pathway (Fig. 8G, H, p < 0.05).

## Discussion

**Fig. 9:**
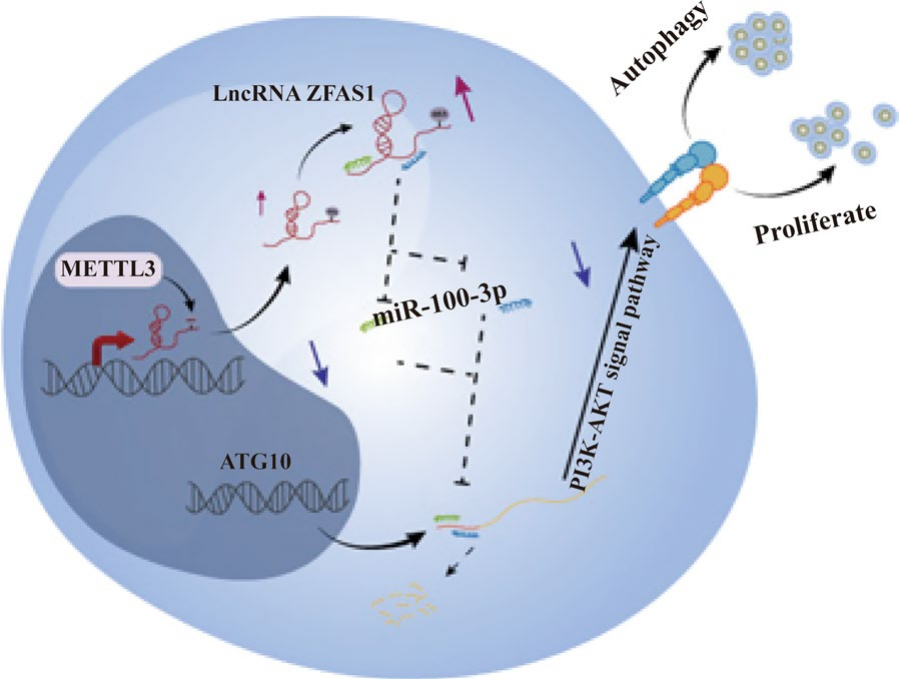
RNA methyltransferase METTL3 affects autophagy and progression of nasopharyngeal carcinoma by regulating the stability of lncRNA ZFAS1. The mechanism route shows that the RNA methyltransferase METTL3 can up-regulate the expression of ZFAS1. Furthermore, ZFAS1 regulates the autophagy level of NPC cells through the PI3K/AKT pathway through miR-100-3p/ATG10, thereby affecting tumor progression

More and more evidences show that the dysregulation of lncRNA plays an important role in the occurrence and development of various types of cancer. We found that ZFAS1 was significantly up-regulated in NPC and this up-regulation was regulated by RNA methyltransferase METTL3. ZFAS1 regulates the expression of ATG10 by competitively adsorbing miR-100-3p to promote the proliferation and metastasis of nasopharyngeal carcinoma cells in vivo and in vitro. In the further discussion of the mechanism, it was found that it regulates the level of autophagy through the PI3K/AKT pathway to promote cancer (such as Fig. 9). The above results suggest that ZFAS1 has carcinogenic effects in the occurrence and development of nasopharyngeal carcinoma, and can be used as a target for nasopharyngeal carcinoma prevention and targeted therapy in the future.

At present, there is evidence that lncRNA is involved in all aspects of tumor formation. In addition, clinically, the current anatomical-based TNM staging system cannot accurately distinguish patients with nasopharyngeal carcinoma [6, 19, 20]. Therefore, the current non-invasive and accurate biomarker screening is an urgent task for clinical treatment of nasopharyngeal carcinoma. In this study, we found that ZFAS1 was significantly higher in tumors than in adjacent tissues by qRT-PCR detection and increased with the increase of stage and degree of malignancy. In addition, we used the TCGA database to analyze the effects of ZFAS1 in other tumors and found that it exists as an oncogene in most tumors and has been partially confirmed [21–23]. These results provide support for our further mechanism discussion and provide a feasible direction for the proposal of biomarkers.

Current reports have shown that m6A modifications pervade the entire transcriptome, accounting for about 0.2% of the total adenosine of cellular RNA [24]. The dynamic reversible modification of m6A installed and deleted by N6 methyltransferases (such as METLL3, METL14 and KIAA1429, etc.) and erasers (such as FTO and ALKBH5) can regulate gene expression and cell fate [25]. Studies have shown that m6A modification is involved in the occurrence and development of a variety of cancers (33–35). The fate of m6A-modified RNA depends on the function of the different proteins that recognize them, which affects stability, translation efficiency, secondary structure, subcellular localization, and alternative polyadenylation [13, 26]. Here, we predicted the possible binding protein of m6A modification and ZFAS1 by means of bioinformatics. Finally, we found that m6A is enriched on ZFAS1 in NPC cells. The modification of m6A in ZFAS1 leads to the improvement of its RNA stability, which may partly explain the up-regulation of ZFAS1 in NPC. This exciting result provides a proven direction for us to explore the cause of the increase in ZFAS1.

Autophagy is a major intracellular degradation system that transports cytoplasmic harmful substances to the lysosome and degrades in the lysosome through the cell membrane transport pathway. The autophagy process can be spontaneous and in dynamic equilibrium, or it can be activated under different stimuli. In addition to cell maintenance, autophagy is also involved in many physiological and pathological conditions, such as aging, apoptosis and cancer [27]. The role of autophagy is complex and differs in different types of cancer. The results of this study show that ZFAS1 can increase the expression of ATG10 by competitively adsorbing miR-100-3p. As an important gene involved in autophagy, ATG10 promotes the increase of autophagy levels [28, 29]. This also explains the phenomenon that the PI3K/AKT pathway is inhibited by ZFAS1 overexpression. A study showed that the increased level of autophagy in the tumor microenvironment is conducive to promoting the absorption of energy by tumor cells, so the increased level of autophagy can promote the development of tumors to a certain extent [30]. Our results show that the up-regulation of ZFAS1 can regulate the autophagy level of tumor cells, and this regulation makes tumor cells have stronger cell viability and migration ability. This phenomenon has also been confirmed in other tumor studies [31]. Although we have verified this result in in vivo and in vitro experiments and concluded that ZFAS1 can affect the process of NPC by regulating the level of autophagy, we need to further design clinical diagnostic experiments to verify ZFAS1 as a biological organism in future studies. The feasibility of the marker is verified.

## Conclusion

Our study verified the cancer-promoting effect of ZFAS1 in NPC and explained part of the reason for its upregulation. In addition, we confirmed that ZFAS1 can regulate the autophagy level of NPC cells through the PI3K/AKT pathway through miR-100-3p/ATG10 to affect tumor progression.

## Supplementary Information

**Additional file 1: Figure S1.** The expression and prognosis of ZFAS1 in different tumors. (A). GEPIA database analyzes the expression of ZFAS1 in different tumors. (B-E). Kaplan–Meier survival analysis of ZFAS1 expression for overall survival.

**Additional file 2: Figure S2.** GEPIA database analyzes the expression of METTL3 in NPC samples. (A). Box plot after data standardization, different colors represent different data sets. (B). PCA results before batch removal for multiple data sets. Different colors represent different data sets. As shown in the schematic diagram, the three data sets are separated without any intersection. (C) PCA results after batch removal, as shown in the schematic diagram Shows the intersection of three data sets, which can be used as a batch of data for subsequent analysis. (D). The expression level of METTL3 in the TCGA database. (E). The expression of METTL3 in different tumor tissue stages in TCGA database. (F). Correlation analysis detects the correlation between the expression of ZFAS1 and METTL3.

**Additional file 3: Figure S3.** miRNA screening. (A). The heat-map of top10 upregulated and down-regulated miRNAs in NPC samples in comparison with corresponding tissues. (B). Venn diagram for screening miRNA. (C). The expression status of miR-100-3p in the TCGA database. (D). QRT-PCR detects the expression of miR-100-3p in different treatment groups. *p<0.05, compared with NC group, #p<0.05, compared with mimics group.

**Additional file 4: Figure S4.** Autophagy and autophagy-related gene screening. (A). Venn diagram screens the target genes of miR-100-3p. (B). The DAVID database performs GO analysis on the target genes of miR-100-3p. (C-D). Detection of mRNA and protein expression of ATG5 and ATG10. (E). The expression of ATG10 in the TCGA database (T=519, N=44). (F-G). Correlation analysis between ZFAS1, ATG10 and miR-100-3p expression.

**Additional file 5.** Clinical sample information and qRT-PCR primer sequence.

## Data Availability

The datasets used and/or analyzed during the current study are available from the corresponding author on reasonable request.

## Abbreviations

NPC: Nasopharyngeal carcinoma
LncRNA: Long non-coding RNA
m6A: N (6)-methyladenosine
ceRNA: Competitive endogenous RNA
miRNA: MicroRNA
TCGA: The Cancer Genome Atlas
HE: Hematoxylin–eosin staining
RIP: Ribonucleoprotein immunoprecipitation
QRT-PCR: Real-time PCR
IHC: Immunohistochemistry
WB: Western blot
mRNA: Messenger RNA

## References

[1] Bray F, Ferlay J, Soerjomataram I (2018). Global cancer statistics 2018: GLOBOCAN estimates of incidence and mortality worldwide for 36 cancers in 185 countries. CA Cancer J Clin.

[2] Chen YP, Chan ATC, Le QT (2019). Nasopharyngeal carcinoma. Lancet.

[3] Wang M, Gu B, Chen X (2019). The function and therapeutic potential of epstein-barr virus-encoded MicroRNAs in cancer. Mol Ther Nucleic Acids.

[4] Campion NJ, Ally M, Jank BJ (2021). The molecular march of primary and recurrent nasopharyngeal carcinoma. Oncogene.

[5] Wen J, Liao J, Liang J (2020). Circular RNA HIPK3: a key circular RNA in a variety of human cancers. Front Oncol.

[6] Guo Z, Wang YH, Xu H (2021). LncRNA linc00312 suppresses radiotherapy resistance by targeting DNA-PKcs and impairing DNA damage repair in nasopharyngeal carcinoma. Cell Death Dis.

[7] Qing X, Tan GL, Liu HW (2020). LINC00669 insulates the JAK/STAT suppressor SOCS1 to promote nasopharyngeal cancer cell proliferation and invasion. J Exp Clin Cancer Res.

[8] Zhou L, Liu R, Liang X (2020). lncRNA RP11–624L4.1 is associated with unfavorable prognosis and promotes proliferation via the CDK4/6-cyclin D1-Rb-E2F1 pathway in NPC. Mol Ther Nucleic Acids.

[9] Miao WJ, Yuan DJ, Zhang GZ (2019). lncRNA CASC2/miR18a5p axis regulates the malignant potential of nasopharyngeal carcinoma by targeting RBBP8. Oncol Rep.

[10] Deng X, Su R, Weng H (2018). RNA N(6)-methyladenosine modification in cancers: current status and perspectives. Cell Res.

[11] Zaccara S, Ries RJ, Jaffrey SR (2019). Reading, writing and erasing mRNA methylation. Nat Rev Mol Cell Biol.

[12] Yang Y, Hsu PJ, Chen YS (2018). Dynamic transcriptomic m(6)A decoration: writers, erasers, readers and functions in RNA metabolism. Cell Res.

[13] Zheng ZQ, Li ZX, Zhou GQ (2019). Long noncoding RNA FAM225A promotes nasopharyngeal carcinoma tumorigenesis and metastasis by acting as ceRNA to sponge miR-590-3p/miR-1275 and upregulate ITGB3. Cancer Res.

[14] Cui Q, Shi H, Ye P (2017). m(6)A RNA methylation regulates the self-renewal and tumorigenesis of glioblastoma stem cells. Cell Rep.

[15] Choe J, Lin S, Zhang W (2018). mRNA circularization by METTL3-eIF3h enhances translation and promotes oncogenesis. Nature.

[16] Xue L, Li J, Lin Y (2021). m(6) A transferase METTL3-induced lncRNA ABHD11-AS1 promotes the Warburg effect of non-small-cell lung cancer. J Cell Physiol.

[17] Li Y, Xiao J, Bai J (2019). Molecular characterization and clinical relevance of m(6)A regulators across 33 cancer types. Mol Cancer.

[18] Xu Z, Han X, Ou D (2020). Targeting PI3K/AKT/mTOR-mediated autophagy for tumor therapy. Appl Microbiol Biotechnol.

[19] Lin L, Liu X, Lv B (2021). Long non-coding RNA MEG3 promotes autophagy and apoptosis of nasopharyngeal carcinoma cells via PTEN up-regulation by binding to microRNA-21. J Cell Mol Med.

[20] Zheng YJ, Zhao JY, Liang TS (2019). Long noncoding RNA SMAD5-AS1 acts as a microRNA-106a-5p sponge to promote epithelial mesenchymal transition in nasopharyngeal carcinoma. FASEB J.

[21] Chen X, Zeng K, Xu M (2018). SP1-induced lncRNA-ZFAS1 contributes to colorectal cancer progression via the miR-150-5p/VEGFA axis. Cell Death Dis.

[22] Gong C, Fan Y, Liu J (2020). METTL14 mediated m6A modification to LncRNA ZFAS1/ RAB22A: a novel therapeutic target for atherosclerosis. Int J Cardiol.

[23] Li Z, Qin X, Bian W (2019). Exosomal lncRNA ZFAS1 regulates esophageal squamous cell carcinoma cell proliferation, invasion, migration and apoptosis via microRNA-124/STAT3 axis. J Exp Clin Cancer Res.

[24] Zhu H, Gan X, Jiang X (2019). ALKBH5 inhibited autophagy of epithelial ovarian cancer through miR-7 and BCL-2. J Exp Clin Cancer Res.

[25] Ban Y, Tan P, Cai J (2020). LNCAROD is stabilized by m6A methylation and promotes cancer progression via forming a ternary complex with HSPA1A and YBX1 in head and neck squamous cell carcinoma. Mol Oncol.

[26] Zhang P, He Q, Lei Y (2018). m(6)A-mediated ZNF750 repression facilitates nasopharyngeal carcinoma progression. Cell Death Dis.

[27] Zhu Q, Zhang Q, Gu M (2020). MIR106A-5p upregulation suppresses autophagy and accelerates malignant phenotype in nasopharyngeal carcinoma. Autophagy.

[28] Catanese A, Olde Heuvel F, Mulaw M (2019). Retinoic acid worsens ATG10-dependent autophagy impairment in TBK1-mutant hiPSC-derived motoneurons through SQSTM1/p62 accumulation. Autophagy.

[29] Huang Q, Liu Y, Zhang S (2020). Autophagy core protein ATG5 is required for elongating spermatid development, sperm individualization and normal fertility in male mice. Autophagy.

[30] Katheder NS, Khezri R, O'Farrell F (2017). Microenvironmental autophagy promotes tumour growth. Nature.

[31] Zhang W, Zhang Y, Xi S (2019). Upregulation of lncRNA HAGLROS enhances the development of nasopharyngeal carcinoma via modulating miR-100/ATG14 axis-mediated PI3K/AKT/mTOR signals. Artif Cells Nanomed Biotechnol.

