# Supplementary figures and images for: The m6A methyltransferase METTL3 affects autophagy and progression of nasopharyngeal carcinoma by regulating the stability of lncRNA ZFAS1
Source: Infect Agent Cancer. 2022 Jan 3;17:1. doi: 10.1186/s13027-021-00411-1 (PMC8722091; doi:10.1186/s13027-021-00411-1)

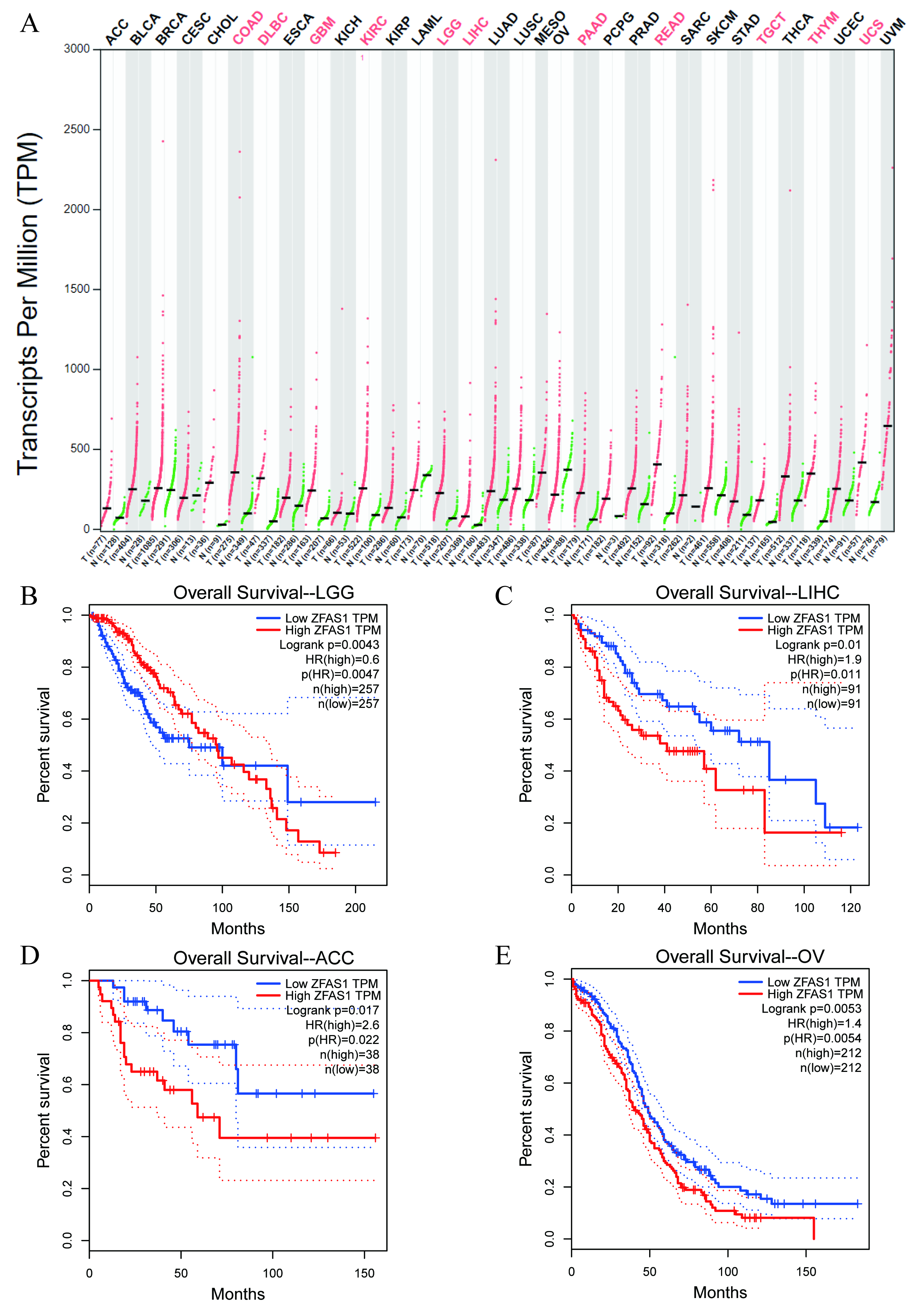

Supplement: Supplementary file 1 — Additional file 1: Figure S1. The expression and prognosis of ZFAS1 in different tumors. (A). GEPIA database analyzes the expression of ZFAS1 in different tumors. (B-E). Kaplan–Meier survival analysis of ZFAS1 expression for overall survival. [file 13027_2021_411_MOESM1_ESM.jpg]

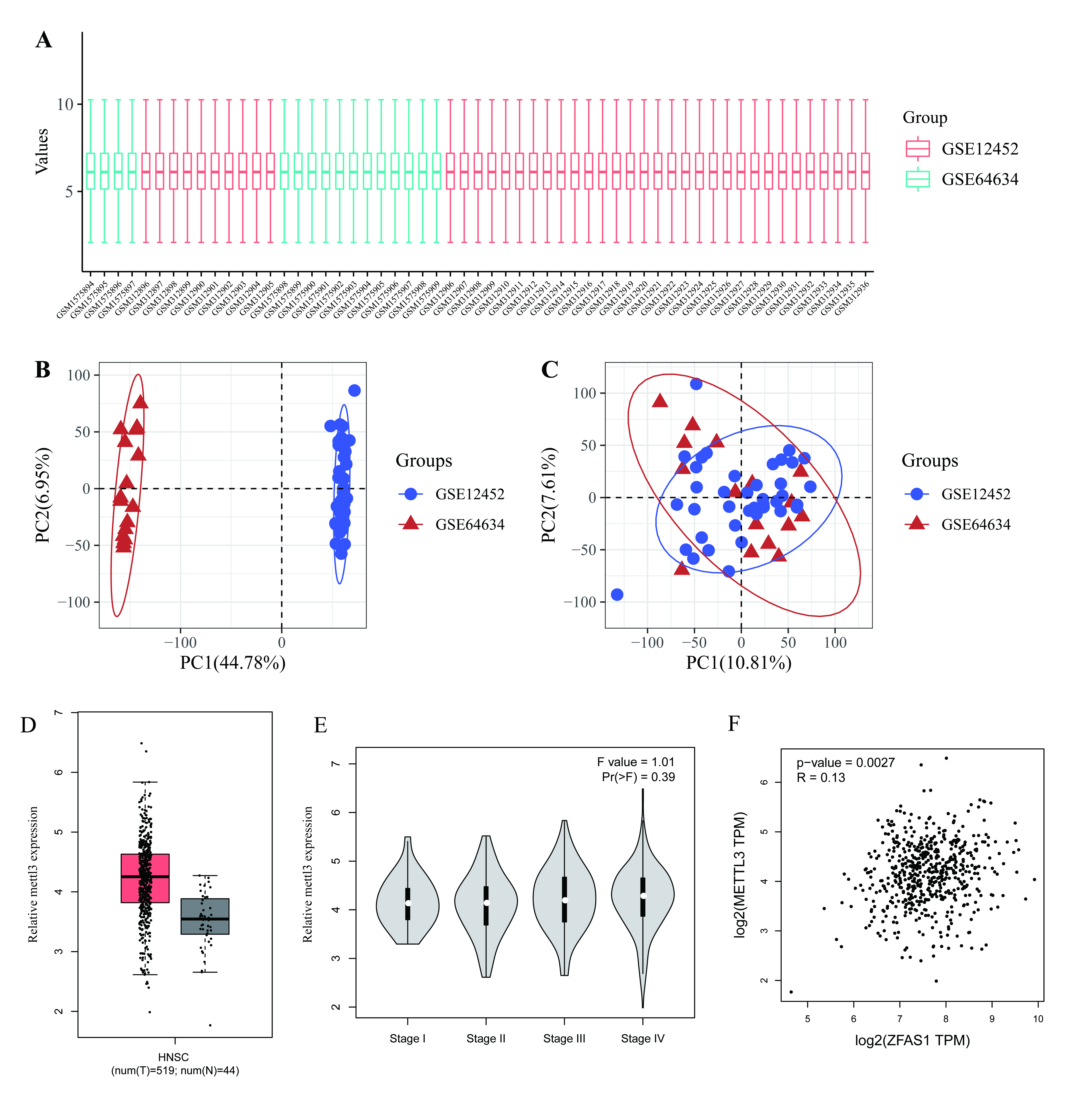

Supplement: Supplementary file 2 — Additional file 2: Figure S2. GEPIA database analyzes the expression of METTL3 in NPC samples. (A). Box plot after data standardization, different colors represent different data sets. (B). PCA results before batch removal for multiple data sets. Different colors represent different data sets. As shown in the schematic diagram, the three data sets are separated without any intersection. (C) PCA results after batch removal, as shown in the schematic diagram Shows the intersection of three data sets, which can be used as a batch of data for subsequent analysis. (D). The expression level of METTL3 in the TCGA database. (E). The expression of METTL3 in different tumor tissue stages in TCGA database. (F). Correlation analysis detects the correlation between the expression of ZFAS1 and METTL3. [file 13027_2021_411_MOESM2_ESM.jpg]

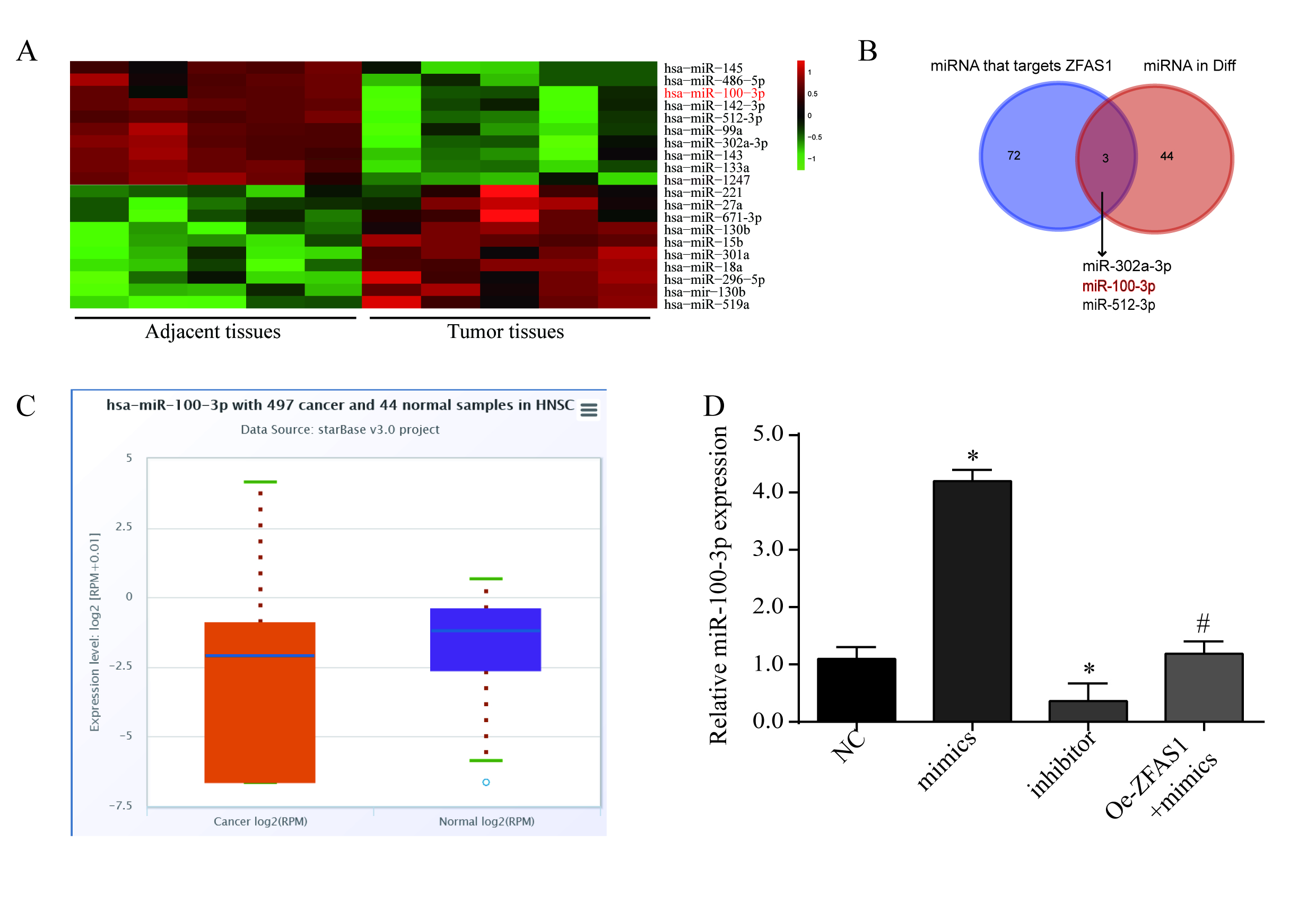

Supplement: Supplementary file 3 — Additional file 3: Figure S3. miRNA screening. (A). The heat-map of top10 upregulated and down-regulated miRNAs in NPC samples in comparison with corresponding tissues. (B). Venn diagram for screening miRNA. (C). The expression status of miR-100-3p in the TCGA database. (D). QRT-PCR detects the expression of miR-100-3p in different treatment groups. *p<0.05, compared with NC group, #p<0.05, compared with mimics group. [file 13027_2021_411_MOESM3_ESM.jpg]

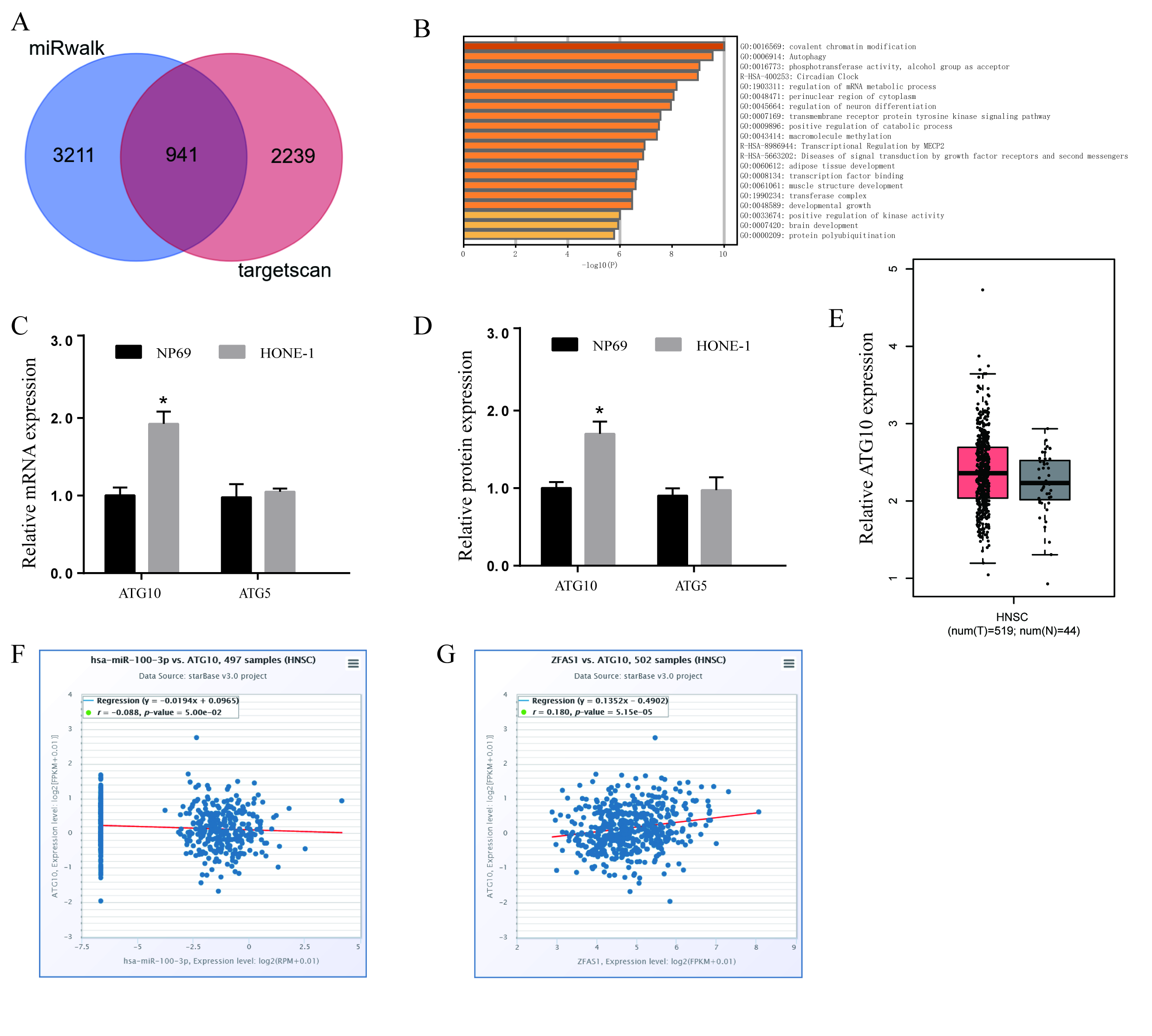

Supplement: Supplementary file 4 — Additional file 4: Figure S4. Autophagy and autophagy-related gene screening. (A). Venn diagram screens the target genes of miR-100-3p. (B). The DAVID database performs GO analysis on the target genes of miR-100-3p. (C-D). Detection of mRNA and protein expression of ATG5 and ATG10. (E). The expression of ATG10 in the TCGA database (T=519, N=44). (F-G). Correlation analysis between ZFAS1, ATG10 and miR-100-3p expression. [file 13027_2021_411_MOESM4_ESM.jpg]
