# Supplementary material for: The m6A methyltransferase METTL3 affects autophagy and progression of nasopharyngeal carcinoma by regulating the stability of lncRNA ZFAS1
Source: Infect Agent Cancer. 2022 Jan 3;17:1. doi: 10.1186/s13027-021-00411-1 (PMC8722091; doi:10.1186/s13027-021-00411-1)
Supplement: Supplementary file 5 — Additional file 5. Clinical sample information and qRT-PCR primer sequence. [file 13027_2021_411_MOESM5_ESM.pdf]

Table S1. The relationship between lncRNA-ZFAS1 expression and clinicopathological characteristics in nasopharyngeal carcinoma

Items	Characteristic	Data (N=53)	ZFAS1 expression		*P* value
			low	high	
Gender	Male	30	14	16	0.7846
	Female	23	12	11	
Age	<50	13	8	5	0.3520
	≥50	40	18	22	
Histological classification	NKC	13	7	6	0.7570
	KSCC	40	19	21	
T stage	T1–T2	28	18	10	**0.0281***
	T3–T4	25	8	17	
TNM stage	I–II	24	16	8	**0.0281***
	III–IV	29	10	19	
Relapse	Yes	43	19	24	0.1751
	No	10	7	3	

Note: *$P < 0.05$ was recognized as a significant difference.

Abbreviations: TNM, tumor node metastasis.

Table S2. Primer sequence

Gene	Primer Sequence
miR-100-3p forward	5'- TCGTTCGCTCAAGCTTGTATCTA -3 '
miR-100-3p reverse	5'- TATGGTTGTTCACCTCTCGTTCAC -3'
U6 forward	5’-CTCGCTTCGGCAGCACATA-3’
U6 reverse	5’-AACGATTCACGAATTTGCGT-3’
ATG10 forward	5’- AGACCATCAAAGGACTGTTCTGA-3’
ATG10 reverse	5’- GGGTAGATGCTCCTAGATGTGA-3’
METTL3 forward	5’- CCCGCGGATATTCACATGGAACTGCC-3’
METTL3 reverse	5’- TGGGGCAGCCATCACAACTGCAAAC-3’
E-cadherin forward	5’-ATTTTTCCCTCGACACCCGAT-3’
E-cadherin reverse	5’-TCCCAGGCGTAGACCAAGA-3’
N-cadherin forward	5’-GAGAGGAAGACCA-GGACTATGA-3’
N-cadherin reverse	5’-CAGTCATCACCACCACCATAC-3’
Vimentin forward	5’-AGTCCACTGAGTACCGGAGAC-3’
Vimentin reverse	5’-CATTTCACGCATCTGGCGTTC-3’
Beclin1 forward	5’-GGCCAATAAGATGGGTCTGA-3’
Beclin1 reverse	5’-GCTGCACACAGTCCAGAAAA-3’
P62 forward	5’-TGTGGAACATGGAGGGAAG-3’
P62 reverse	5’-TGTGCCTGTGCTGGAACTTTC-3’
LC3 forward	5’-TTGGTCAAGATCATCCGGC-3’
LC3 reverse	5’-GCTCACCATGCTGTGCTGG-3’
ZFAS1 forward	5’- GCTATTGTCCTGCCCGTTAG -3’
ZFAS1 reverse	5’- TCGTCAGGAGATCGAAGGTT -3’
GAPDH forward	5’-GAAGGTGAAGGTCGGAGTC-3’
GAPDH reverse	5’-GAAGATGGTGATGGGATTTC-3’
